# Transmembrane p24 trafficking protein 2 regulates inflammation through the TLR4/NF-κB signaling pathway in lung adenocarcinoma

**DOI:** 10.1186/s12957-021-02477-y

**Published:** 2022-02-08

**Authors:** Longhua Feng, Pengjiang Cheng, Zhengyun Feng, Xiaoyu Zhang

**Affiliations:** 1 Department of Respiratory, Qianjiang Central Hospital of Chongqing, Chongqing, 409000 People’s Republic of China; 2 Department of Intensive Care Unit, Qianjiang Central Hospital of Chongqing, No.63, Chengxijiu Road, Qianjiang District, Chongqing, 409000 People’s Republic of China

**Keywords:** TMED2, Lung adenocarcinoma, Inflammation, TLR4, NF-κB

## Abstract

**Background:**

To investigate the role of transmembrane p24 trafficking protein 2 (TMED2) in lung adenocarcinoma (LUAD) and determine whether TMED2 knockdown could inhibit LUAD in vitro and in vivo.

**Methods:**

TIMER2.0, Kaplan-Meier plotter, gene set enrichment analysis (GSEA), Target Gene, and pan-cancer systems were used to predict the potential function of TMED2. Western blotting and immunohistochemistry were performed to analyze TMED2 expression in different tissues or cell lines. The proliferation, development, and apoptosis of LUAD were observed using a lentivirus-mediated TMED2 knockdown. Bioinformatics and western blot analysis of TMED2 against inflammation via the TLR4/NF-κB signaling pathway were conducted.

**Results:**

TMED2 expression in LUAD tumor tissues was higher than that in normal tissues and positively correlated with poor survival in lung cancer and negatively correlated with apoptosis in LUAD. The expression of TMED2 was higher in tumors or HCC827 cells. TMED2 knockdown inhibited LUAD development in vitro and in vivo and increased the levels of inflammatory factors via the TLR4/NF-κB signaling pathway. TMED2 was correlated with TME, immune score, TME-associated immune cells, their target markers, and some mechanisms and pathways, as determined using the TIMER2.0, GO, and KEGG assays.

**Conclusions:**

TMED2 may regulate inflammation in LUAD through the TLR4/NF-κB signaling pathway and enhance the proliferation, development, and prognosis of LUAD by regulating inflammation, which provide a new strategy for treating LUAD by regulating inflammation.

## Introduction

Lung adenocarcinoma (LUAD) is the most common type of non-small cell lung cancer (NSCLC), which is a devastating disease characterized by poor patient survival [1]. Immunotherapy-based approaches are the standard first-line treatments for metastatic lung cancer, whereas consolidation chemoradiotherapy is the standard of care in locally advanced disease [2]. Immune checkpoint blockade therapies have revolutionized the treatment of LUAD [3]. The past decade has witnessed the translation of knowledge derived from preclinical studies of antitumor immunity into approved immunotherapies for cancer [4]. Therefore, analyses of the actual inflammation markers of the cell of origin and the regulation of its growth will be useful for the treatment, prophylaxis, and prevention of LUAD. In contrast, the successful implementation of treatments that target LUAD-associated inflammation is still awaited.

TLR4 is a key regulator of both innate and acquired immunity and has been linked to the development of various cancers [5]. Recently, TLR4 was reported to be the functional receptor of resistin in cell migration and invasion in lung cancer, whereas nuclear factor (NF)-κB is the intracellular downstream effector mediating resistin-induced migration and invasion [6]. Therefore, identifying the key inflammation-regulating factors affecting the TLR4/NF-κB signaling pathway may be a vital step in LUAD treatment.

Transmembrane p24 trafficking protein 2 (*TMED2*) is a protein-coding gene associated with cardiomyopathy that is involved in vesicular protein trafficking [7, 8]. TMED2 was recently reported to be involved in multiple myeloma, breast cancer, hepatocellular carcinoma, and choriocarcinoma [9–12]. However, the mechanism underlying the role of TMED2 in inflammation via the TLR4/NF-κB signaling pathway in LUAD is not yet fully understood.

The present study aimed to investigate the role of TMED2 in LUAD. Lentivirus-mediated TMED2 knockdown (using sh-TMED2) was performed to inhibit inflammation and tumor development in LUAD both in vivo and in vitro. The present findings indicate that TMED2 could regulate inflammation in LUAD via the TLR4/NF-κB signaling pathway. These results may then provide novel strategies for LUAD treatment.

## Materials and methods

### Human tumor specimens

Nineteen patients pathologically diagnosed with LUAD at the Department of Pathology of Qianjiang Central Hospital of Chongqing from May 2019 to May 2021 were enrolled in the study, and adjacent para-cancerous normal alveolar tissues from patients were obtained as controls. All patients provided informed consent, and the experimental design was approved by the ethics committee of Qianjiang Central Hospital of Chongqing (NO.2018QCHC0032).

### Bioinformatics analysis

TIMER2.0 (http://timer.cistrome.org/) was used to analyze the expression of TMED2 in different cancers, as described previously [13]. A survival analysis of the LUAD patients in the TMED2-low and high expression groups was performed using Kaplan-Meier plotter (http://kmplot.com/analysis/index.php?p=service&cancer=lung), as described previously [14]. The pan-cancer assay was downloaded from The Cancer Genome Atlas (TCGA) database, as described previously [15]. Gene set enrichment analysis (GSEA) (4.0.3) was used to analyze LUAD-related genes from TCGA, as described previously [16]. A target gene assay was performed to analyze the related genes for mutation and prognosis using a website (https://www.mutarget.com/analysis?type=target), as described previously [14, 17].

### Cell lines and cell culture

Human alveolar epithelium (HAE, normal), as well as A549, HCC827, HCL-H1299, and HCL-H524 (NSCLC) cell lines, was used in this study. Cells were maintained in Ham’s F12K medium (Life Technologies, MA, USA) and RPMI-1640 medium (Thermo Fisher Scientific, Inc., MA, USA), as described previously [18].

### Animals

Healthy 4-week-old male Balb/c mice were supplied by Liaoning Changsheng Biotechnology Co., Ltd. (Shenyang, Liaoning, China). The mice were housed with food and water under specific pathogen-free (SPF) conditions. Mice were reared in strict accordance with the Guide for the Care and Use of Laboratory Animals (NIH Publication No. 85-23, revised 1996), and the experimental design was approved by the Ethics Committee of Qianjiang Central Hospital of Chongqing (NO.2019QCHC0302).

### Cell lines and antibodies

The cell lines used were as follows: HAE (CP-H209, Procell, Wuhan, China), A549 (CL-0016, Procell), HCC827 (CL-0094, Procell), NCL-H1299 (CL-0165, Procell), and NCL-H524 (CL-0403, Procell).

The following antibodies were used: anti-TMED2 (ab251705, Abcam, Cambridge, USA), anti-Ki-67 (ab15580, Abcam), anti-CEA (ab207718, Abcam), anti-NSE (ab180943, Abcam), anti-EGFR (ab200828, Abcam), anti-TLR4 (ab13556, Abcam), anti-NF-κB-p-p65 (BM3940, Boster, Wuhan, China), anti-IL-1β (ab2105, Abcam); anti-IL-18 (ab207323, Abcam); and anti-β-actin (M01263-2, Boster). The secondary antibodies used were anti-rabbit IgG (AS014, ABclonal, Wuhan, China) and anti-mouse IgG (H+L) (AS003, ABclonal).

### Lentivirus-mediated TMED2 knockdown

TMED2-knockdown lentiviruses (sh-TMED2) and control lentiviruses were designed and chemically synthesized by GenePharma Corporation (Shanghai, China) and stored at − 80 °C until use. Lentiviral transduction was performed as previously established [19]. The sequences of the TMED2-knockdown shRNAs sequences were as follows: sh-TMED2-122, 5′-gctctcctggccacggtctc-3′; sh-TMED2-241, 5′-cggcttcctggacatcgacg-3′; sh-TMED2-663, 5′-gacagatctactacctgaag-3′; Negative Control, 5′-ttcctgatgatcccaactca-3′ and control-shTMED2, 5′-gaattcactgtttaccaaac-3′.

Mice were then randomly divided into four groups (*n* = 6 in each group): (1) con group: untreated HCC827 cells or mice administered with HCC827 cells; (2) sh-TMED2 group: HCC827 cells treated with sh-TMED2 lentivirus or mice subjected to an injection of HCC827 cells treated with sh-TMED2 lentivirus; (3) control-shTMED2 group: HCC827 cells treated with TMED2 control lentivirus or mice subjected to an injection of HCC827 cells treated with TMED2 control lentivirus; and (4) Spa + sh-TMED2 group: HCC827 cells treated with sh-TMED2 or mice injected with HCC827 cells treated with sh-TMED2 or sparstolonin B (10 μg in vitro or 10 mg/kg in vivo).

### Subcutaneous xenograft mouse model

The mice were subcutaneously injected with HCC827 cells. After 5 days, the vital signs and inoculation sites of the mice were observed as described previously [20]. The mice were euthanized when they exhibited rapid weight loss (> 20%) and showed signs of deteriorating health due to metastatic burden, such as hunching, dehydration, and labored breathing.

### Western blotting

A total of 50 μg of protein from each sample was subjected to 12% SDS-polyacrylamide gel electrophoresis and transferred to nitrocellulose membranes as described previously [21]. The membranes were developed using a ChemiDoc™ Touch Imaging System and quantified using Image Lab software (version 3.0; Bio-Rad, CA, USA).

### Immunohistochemical staining

For immunohistochemical staining, 4-μm-thick tissue sections were incubated with primary antibodies against TMED2 and Ki-67 at 4 °C overnight. The sections were then incubated with the corresponding secondary antibodies at room temperature for 2 h, as described previously [22]. The signals were measured using a commercial streptavidin-biotin-peroxidase staining kit (Goldenbridge Biotechnology Co., China).

### Propidium iodide (PI) staining

Cells were subjected to a PI-Hoechst assay (40755ES64, Qcbio Science&Technologies Co., Ltd, Shanghai, China) as described previously [23]. Images were acquired using a Nikon Eclipse Ti inverted microscope (TE2000; Nikon, Tokyo, Japan).

### Terminal deoxynucleotidyl transferase dUTP nick end labeling (TUNEL) staining

Tissue fractions from each group were analyzed using a TUNEL assay (ab66108, Abcam, Cambridge, UK), as described previously [24]. Images were captured using a fluorescence microscope at × 400 magnification.

### 3-(4,5)-dimethylthiahiazo (-z-y1)-3,5-di- phenytetrazoliumromide (MTT) assay

After stirring the solution for 30 min, they were filter-sterilized using a microporous filter (diameter, 0.22 μm), followed by storage at 4 °C until use, as described previously [25].

### Statistical analysis

Data are expressed as the means ± standard deviations and were analyzed via one-way or two-way ANOVA using GraphPad Prism 6.0 (GraphPad Software, San Diego, USA) and SPSS 18.0 (IBM, New York, USA) software. Multiple comparisons were performed using the Holm-Sidak test. Statistical significance was set at *P* < 0.05.

## Results

### Bioinformatics analysis of TMED2 in LUAD using TIMER2.0, Kaplan-Meier plotter, and GSEA

**Fig. 1:**
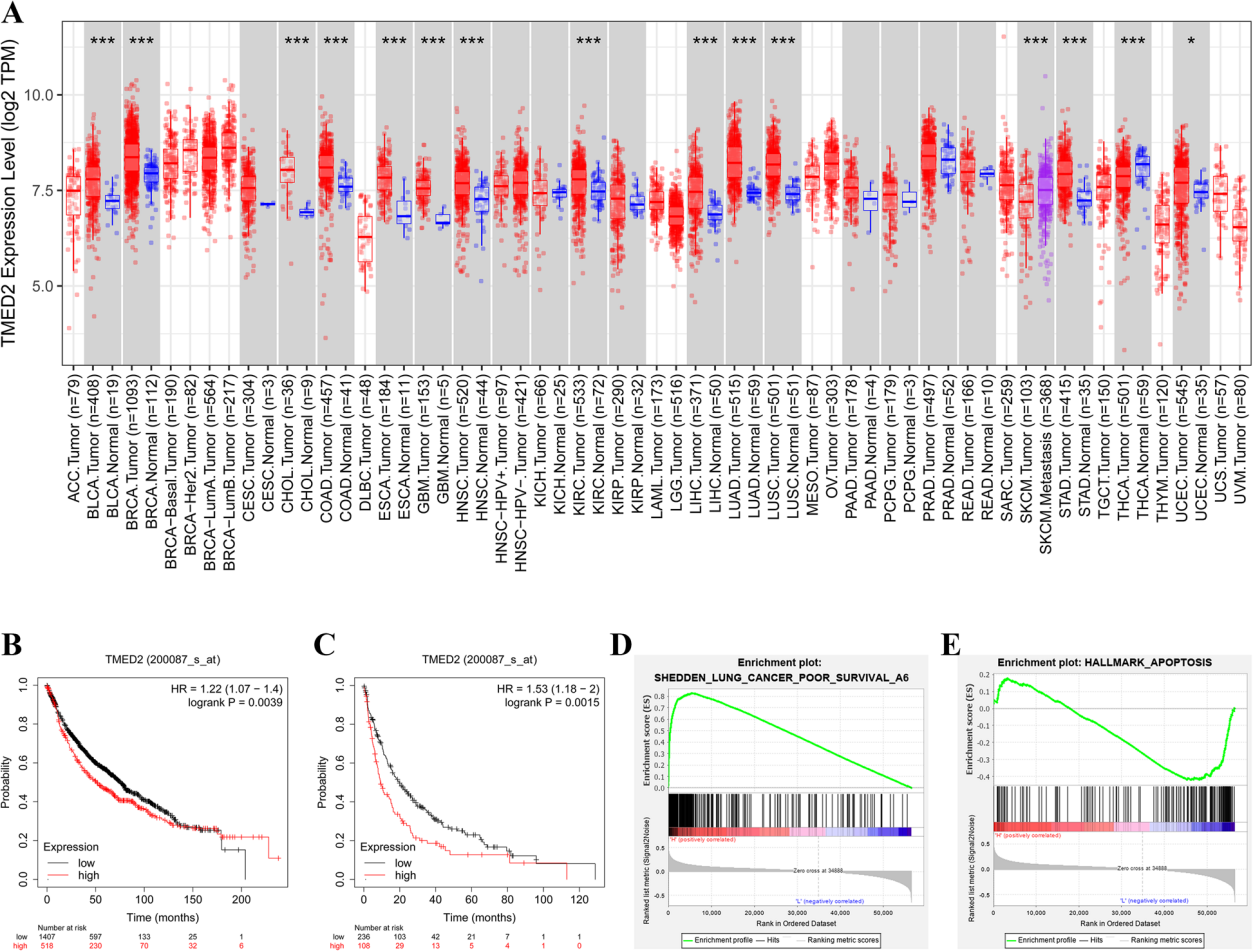
**A** Expression of TMED2 in different cancers, as determined via the TIMER2.0 system. **B** OS and **C** PPS analyses between LUAD patients with high and low TMED2 expression, as determined using the Kaplan-Meier plotter system. **D** GSEA of TMED2 with lung cancer poor survival. **E** GSEA of TMED2 with apoptosis in LUAD

The predicted role of TMED2 in LUAD was detected using TIMER2.0, Kaplan-Meier plotter, and GSEA, as shown in Fig. 1. TMED2 expression was quantified in a series of common cancers via TIMER2.0, and it was found that the level of TMED2 in LUAD tumor tissues was approximately 1.25 log_2_TPM higher than that in normal tissues (*P* < 0.05). In addition, for the analyses of overall survival (OS) and post-progressive survival (PPS) using the Kaplan-Meier plotter system, given their high level of TMED2 expression (Fig. 1B, C), LUAD patients were predicted to have a worse outcome (*P* < 0.05). Furthermore, the GSEA showed that TMED2 in LUAD was positively correlated with poor survival and was negatively correlated with apoptosis (Fig. 1D, E).

### TMED2 expression in tumor tissues and different LUAD cell lines

**Fig. 2:**
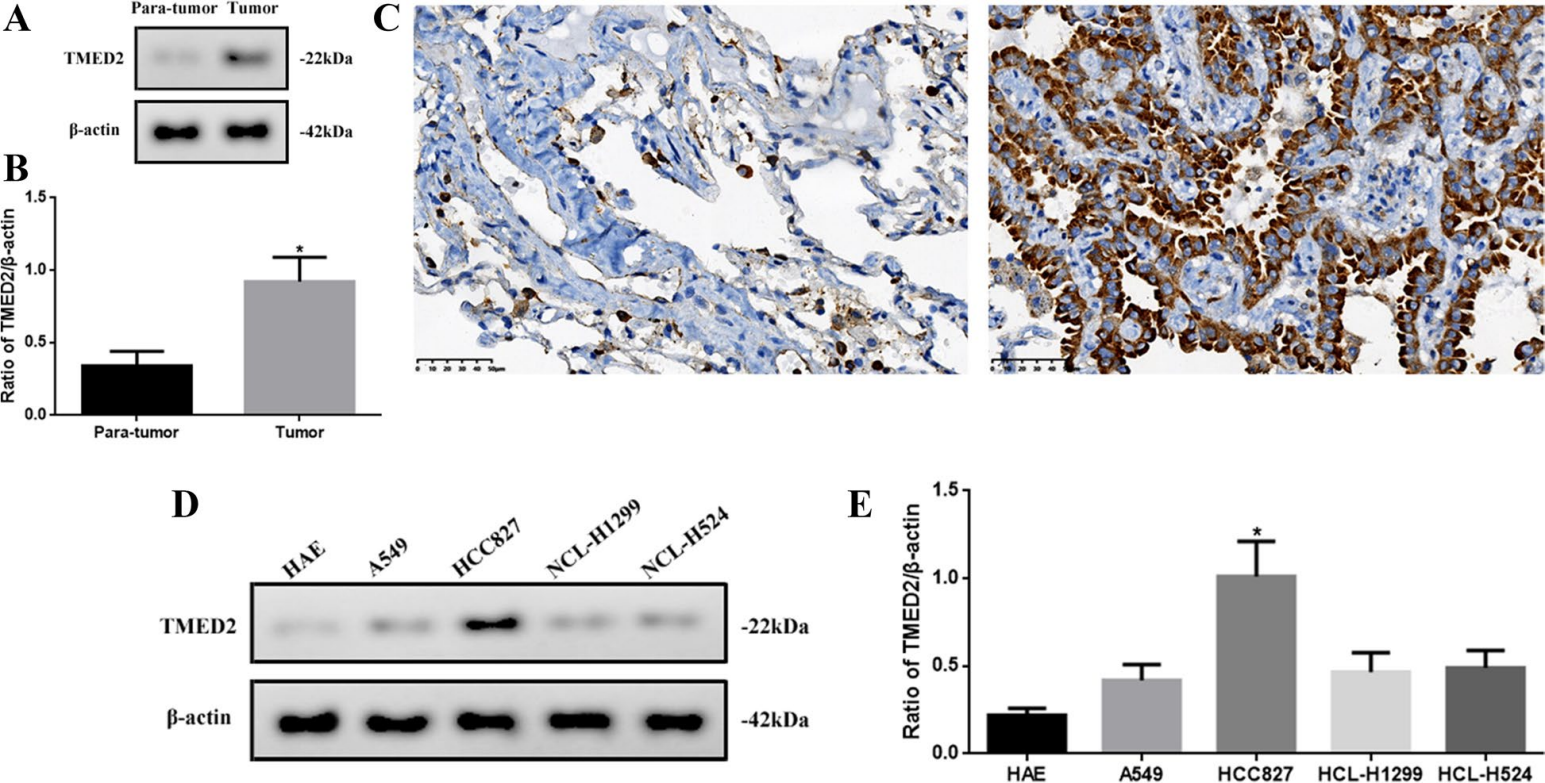
**A** Western blot assay of TMED2 expression in para-tumor and tumor tissues. **B** Quantification of TMED2 expression via western blot and **C** IHC in para-tumor tissue and tumor tissue (× 400). **D** Western blot assay of TMED2 expression in HAE, A549, HCC827, HCL-H1299, and HCL-H524 cells. **E** Quantification of TMED2 protein expression in different cell lines. Protein levels were normalized to β-actin (tumor vs. para-tumor, or HCC827 vs. other cells; *P < 0.05, *n* = 6 per group; all data are represented as the mean ± standard deviations)

Bioinformatics analysis showed that TMED2 may be associated with LUAD; therefore, we detected the TMED2 expression levels in tumor tissues or LUAD cell lines (Fig. 2). The expression of TMED2 in tumor tissues was significantly higher than that in para-tumor tissues (*P* < 0.05, Fig. 2A, B). Similar to the results of immunohistochemistry (IHC) (Fig. 2C), the approximate localization of TMED2 was determined to be in the cytoplasm and cell membrane. In addition, the expression level of TMED2 in HCC827 cells was significantly higher (*P* < 0.05), compared with the other cells (Fig. 2D, E). Therefore, we chose HCC827 as the LUAD model cell line for the follow-up experiments in our study.

### Knocking down TMED2 inhibited the development of LUAD in vitro

**Fig. 3:**
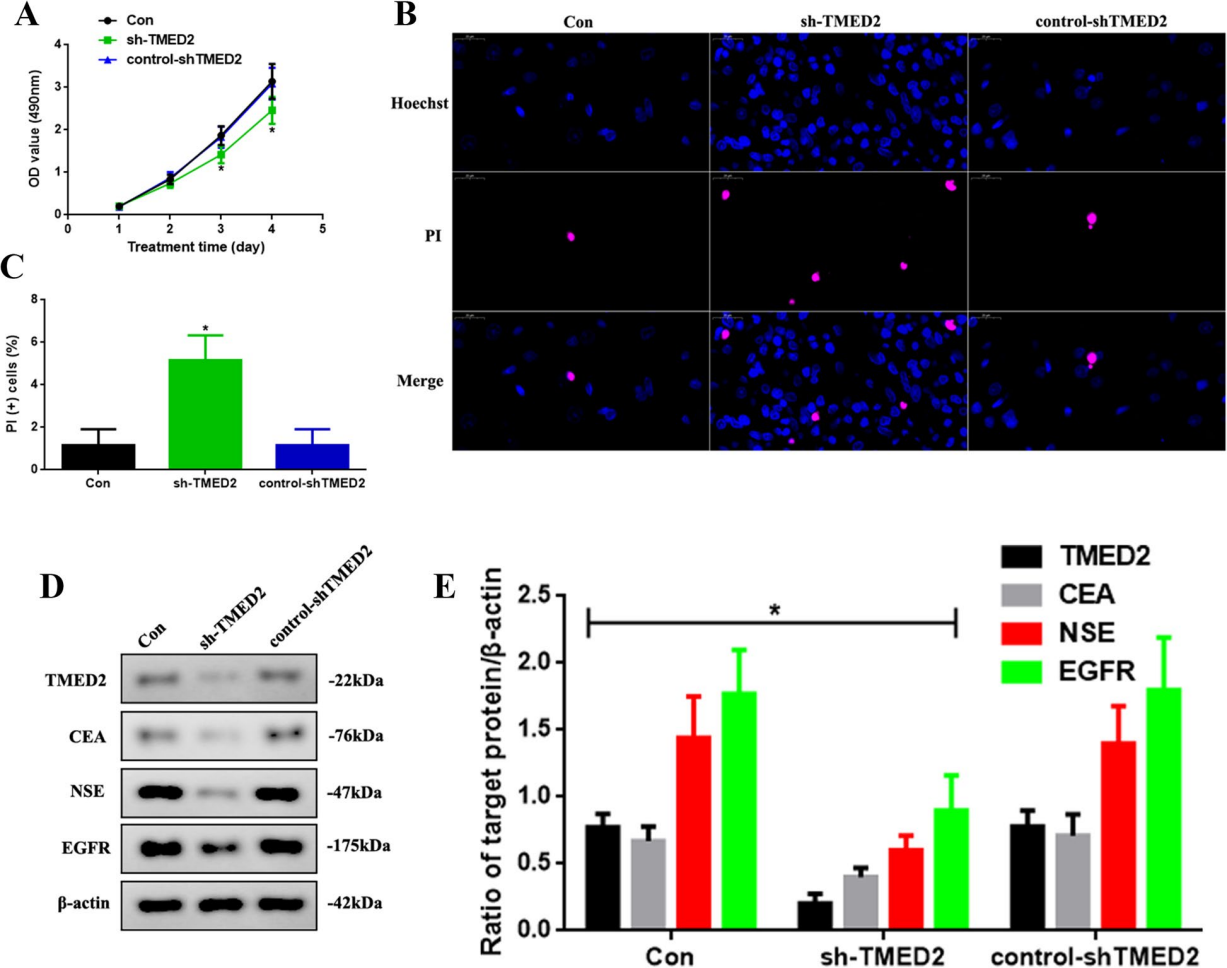
**A** Proliferation assay of the Con, sh-TMED2, and control-shTMED2 group after 4 days of treatment. **B** PI-Hoechst staining assay (× 800) and **C** quantification of PI(+) cells. **D** Western blot assay of TMED2, CEA, NSE, and EGFR expression in vitro. **E** Quantification of CEA, NSE, and EGFR expression. Protein levels were normalized to β-actin. (sh-TMED2 vs. other groups, *P < 0.05, *n* = 6 per group)

Since TMED2 might be correlated with the progression of LUAD, as suggested in our previous results, we attempted to decrease the expression of TMED2 via lentivirus-mediated knockdown (sh-TMED2), as shown in Fig. 3. We observed the proliferation of Con, sh-TMED2, and control-shTMED2 HCC827 cells (Fig. 3A). The OD value at 3 and 4 days, as detected via MTT in the sh-TMED2 group, was decreased (*P* < 0.05). To investigate the apoptotic function of TMED2, we used PI-Hoechst staining (Fig. 3B) and found that apoptosis was increased in the sh-TMED2 group (Fig. 3C, *P* < 0.05). In addition, we detected the levels of tumor biomarkers in LUAD (Fig. 3D) and found that the levels of TMED2, CEA, NSE, and EGFR in the sh-TMED2 group were reduced (*P* < 0.05, Fig. 3E).

### Knocking down TMED2 inhibited the development of LUAD in vivo

**Fig. 4:**
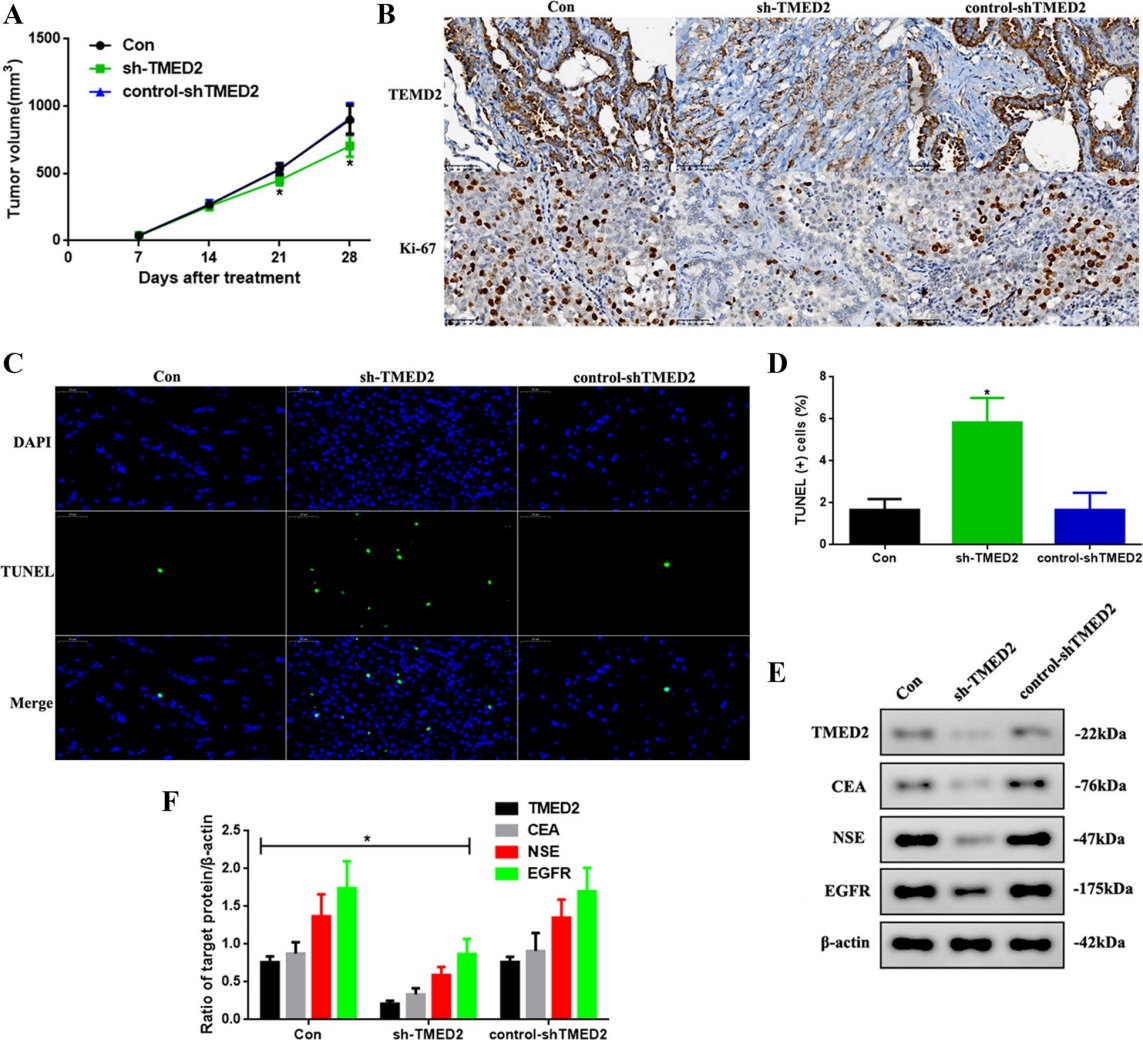
**A** Tumor volume in the Con, sh-TMED2, and control-shTMED2 mice after 28 days. **B** IHC for TMED2 and determination of the Ki-67 index. **C** TUNEL-DAPI staining assay (× 400) and **D** quantification of TUNEL(+) cells. **E** Western blot assay of CEA, NSE, and EGFR expression in vivo. **F** Quantification of TMED2, CEA, NSE, and EGFR expression. Protein levels were normalized to β-actin. (sh-TMED2 vs. other groups, *P < 0.05, *n* = 6 per group)

Knocking down TMED2 could inhibit the development of LUAD in vitro; thus, we determined if this would be consistent in vivo (Fig. 4). First, we observed the tumor volumes of the mice treated with the different cell constructs. The tumor volumes at days 21 and 28 in the sh-TMED2 mice were significantly decreased (*P* < 0.05, Fig. 4A). Next, the expression of TMED2 and the proliferation index were detected via IHC (Fig. 4B), and it was found that TMED2 and Ki-67 levels were reduced after TMED2 knockdown. Further, to investigate the apoptotic function of TMED2, we used TUNEL staining (Fig. 4C) and found that the incidence of apoptosis in sh-TMED2 mice was increased (Fig. 4D, *P* < 0.05). Finally, we also detected the levels of tumor biomarker levels in LUAD (Fig. 4E) and found that the expression of TMED2, CEA, NSE, and EGFR in sh-TMED2 mice was reduced (*P* < 0.05, Fig. 4F), consistent with the in vitro results.

### GSEA of TMED2 with inflammation, TLR4 and NF-κB

**Fig. 5:**
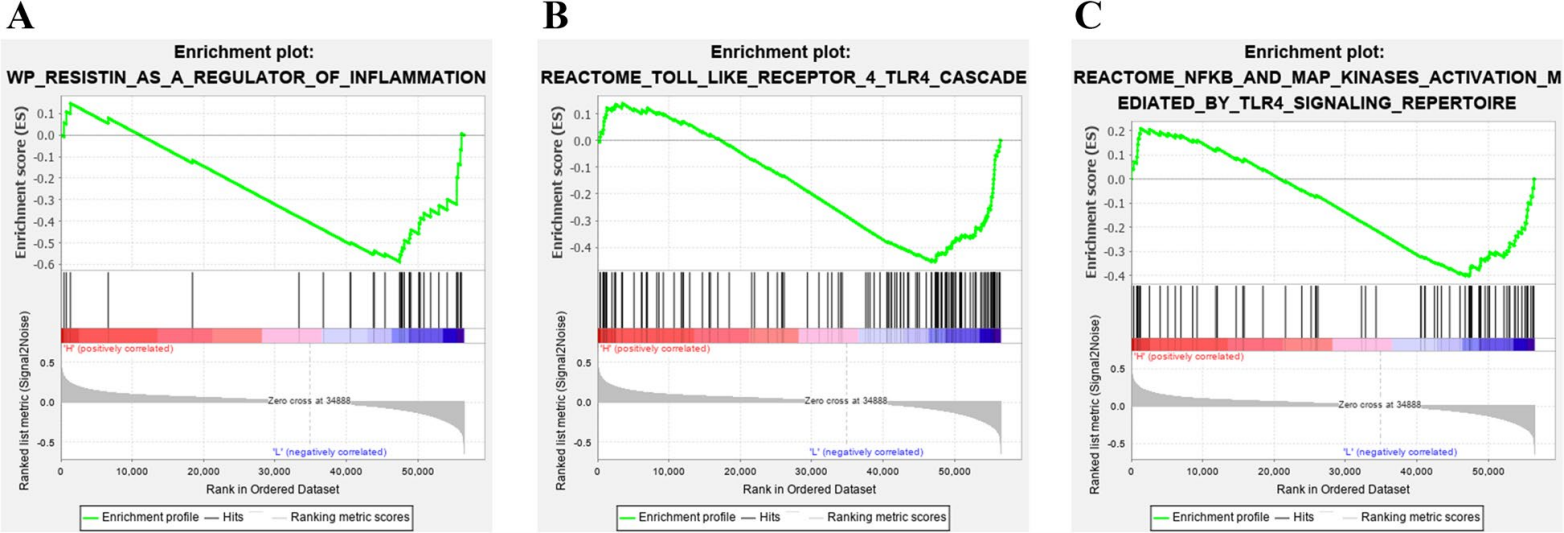
GSEA of TMED2 with **A** inflammation, **B** TLR4, and **C** NF-κB in LUAD

To investigate the potential mechanism of TMED2 in regulating the progression of LUAD, we performed GSEA (Fig. 5). The results showed that TMED2 was negatively correlated with inflammation, TLR4, and NF-κB in LUAD (Fig. 5A–C).

### TMED2 knockdown increased inflammation that was regulated through the TLR4/NF-κB signaling pathway in vitro and in vivo

**Fig. 6:**
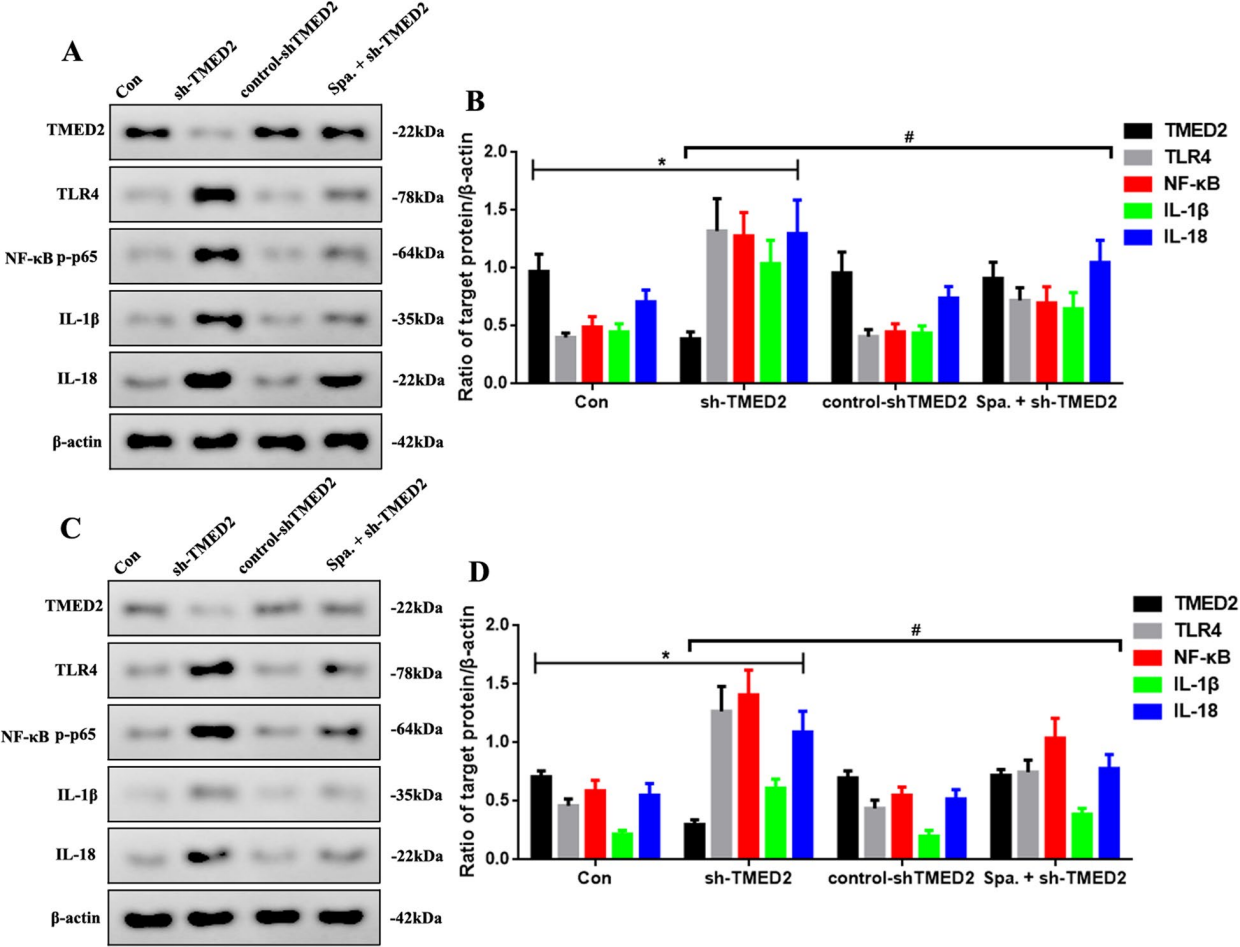
**A** Western blot assay of TMED2, TLR4, NF-κB-p-p65, IL-1β, and IL-18 expression in the Con, sh-TMED2, control-shTMED2, and Spa. + sh-TMED2 groups in vitro. **B** Quantification of TMED2, TLR4, NF-κB-p-p65, IL-1β, and IL-18 protein expression. **C** Western blot assay of TMED2, TLR4, NF-κB-p-p65, IL-1β, and IL-18 expression in vivo. **D** Quantification of TMED2, TLR4, NF-κB-p-p65, IL-1β, and IL-18 protein expression. Protein levels were normalized to β-actin. (sh-TMED2 vs. Con group, *P < 0.05; Spa. + sh-TMED2 vs. sh-TMED2 group, #*P* < 0.05, *n* = 6 per group)

To further confirm the tumor-inhibition effect of TMED2 and whether it was mediated through the TLR4/NF-κB signaling pathway, we used an activator of TLR4 (sparstolonin B) to boost TLR4 expression and observed the changes in the downstream inflammatory factors using western blotting (Fig. 6). As shown in Fig. 6A, sparstolonin B increased the levels of TLR4, NF-κB-p-p65, IL-1β, and IL-18 after sh-TMED2 administration (*P* < 0.05), compared to the sh-TMED2 group (Fig. 6B) in vitro. Furthermore, sparstolonin B did not significantly alter TMED2 levels in vitro. Similar outcomes were observed in vivo*,* as shown in Fig. 6C, D.

### Bioinformatics analysis of TMED2 in the TME, immune score, TME-associated immune cells, and their target markers in LUAD assessed using a pan-cancer system and TIMER2.0

**Fig. 7:**
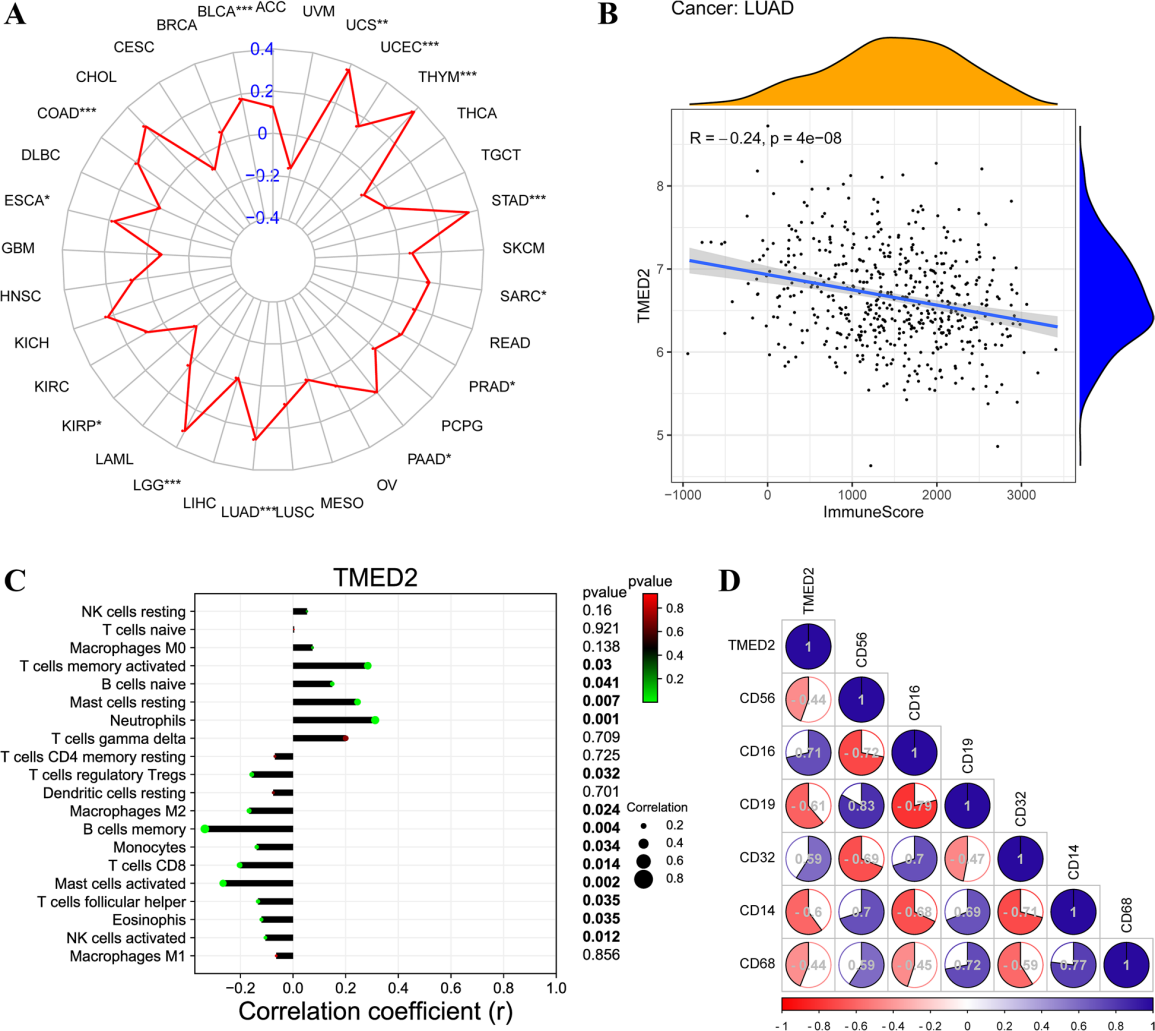
**A** Pan-cancer assay of TMED2 in the TME. **B** Correlation analysis between TMED2 and immune scores in LUAD. **C** Correlation analysis between TMED2 and TME-associated immune cells. **D** Correlation analysis between TMED2 and the target markers of TME-associated immune cells

Since TMED2 was predicted to be an immunoregulatory gene in LUAD, we predicted TMED2 in the TME, immune score, TME-associated immune cells, and their target markers in LUAD. TMED2 was negatively correlated with the TME and was positively correlated with the immune score (Fig. 7A, B). Further, TMED2 was negatively correlated with regulatory T cells (Tregs), M2 macrophages, memory B cells, monocytes, CD8+ T cells, activated mast cells, follicular helper T cells, eosinophils, and NK cells and was positively correlated with activated memory T cells, naïve B cells, resting mast cells, and neutrophils (Fig. 7C). Furthermore, TMED2 was negatively correlated with CD56, CD19, CD14, and CD68, and was positively correlated with CD16 and CD32 (Fig. 7D).

### Bioinformatics analysis of TMED2 using Gene Ontology (GO) and Kyoto Encyclopedia of Genes and Genomes (KEGG) in pan-cancer

**Fig. 8:**
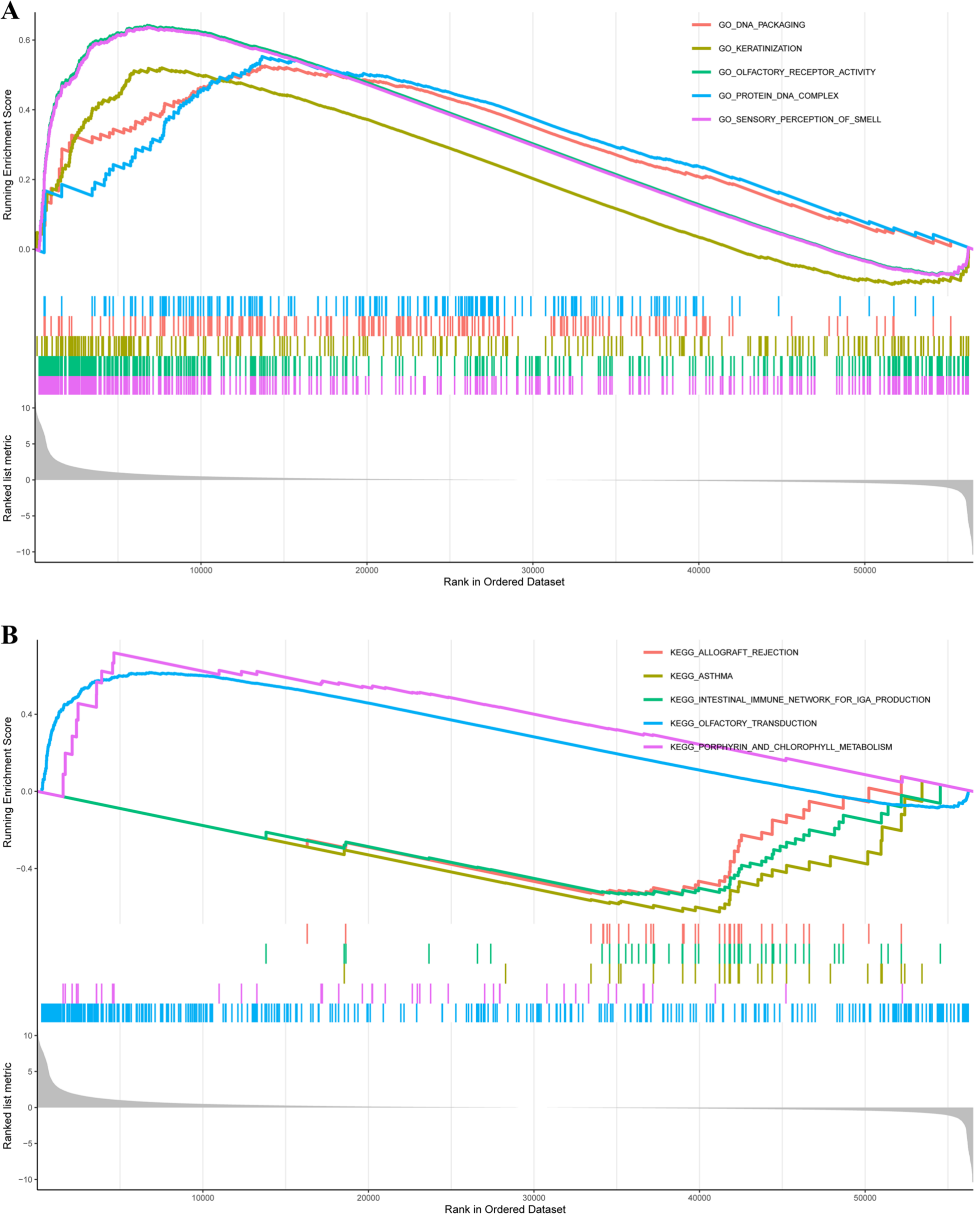
**A** GO analysis of TMED2 using a pan-cancer assay. **B** KEGG analysis of TMED2

In the GO analysis, we found that TMED2 was positively correlated with DNA packaging, keratinization, olfactory receptor activity, protein DNA complex, and sensory perception of smell (Fig. 8A). In the KEGG analysis, TMED2 was negatively correlated with olfactory transduction, porphyrin, and chlorophyll metabolism and was positively correlated with allograft rejection, asthma, and the intestinal immune network for IgA production (Fig. 8B).

### Target gene analysis of TMED2 in LUAD patients

**Fig. 9:**
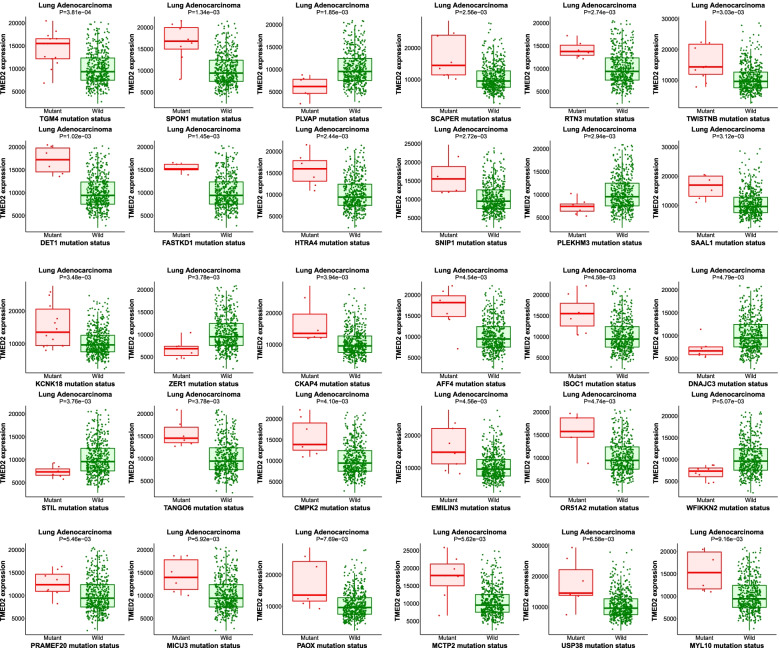
**A** Association among TMED2 and TGM4, SPON1, PLVAP, SCAPER, RTN3, TWISTNB, DET1, FASTKD1, HTRA4, SNIP1, PLEKHM3, SAAL1, KCNK18, ZER1, CKAP4, AFF4, ISOC1, DNAJC3, STIL, TANGO6, CMPK2, EMILIN3, OR51A2, WFIKKN2, PRAMEF20, MICU3, PAOX, MCTP2, and USP38 mutants and LUAD patients with wild-type mutation, assessed using the Target Gene system

The results of the target gene analysis of TMED2 in LUAD are shown in Fig. 9. At higher expression levels of TMED2 in LUAD patients, mutations in TGM4, SPON1, PLVAP, SCAPER, RTN3, TWISTNB, DET1, FASTKD1, HTRA4, SNIP1, PLEKHM3, SAAL1, KCNK18, ZER1, CKAP4, AFF4, ISOC1, DNAJC3, STIL, TANGO6, CMPK2, EMILIN3, OR51A2, WFIKKN2, PRAMEF20, MICU3, PAOX, MCTP2, and USP38 were found. These results suggest that TMED2 is related to these gene mutations and is involved in LUAD prognosis.

**Fig. 10:**
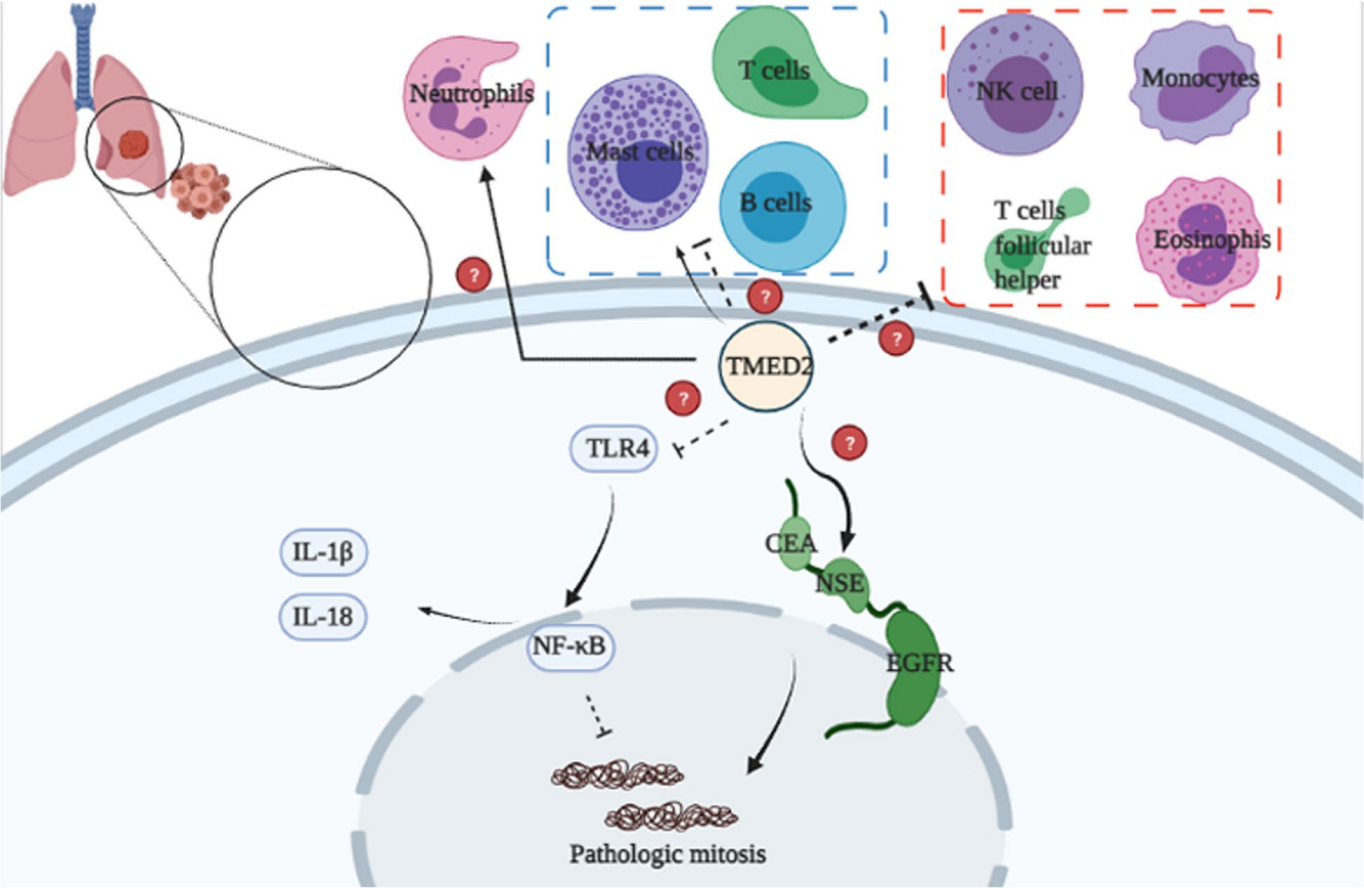
The potential mechanisms of TMED2-mediated inflammation regulation in LUAD. TMED2 may regulate inflammation via the TLR4/NF-κB signaling pathway, decreasing the levels of inflammatory factors in tumor cell and then increasing their pathological mitosis

The potential mechanisms of TMED2-induced inflammation in LUAD are shown in Fig. 10. TMED2 probably regulates inflammation via the TLR4/NF-κB signaling pathway, enhancing the proliferation and development of LUAD cells, and is involved in prognosis. Furthermore, TMED2 may regulate TME-associated immune cells in LUAD.

## Discussion

LUAD is the most common histologic subtype of NSCLC, a fatal disease with a low 5-year survival rate [1, 26]. As it exhibits a prolonged asymptomatic condition and a high recurrence rate, its treatment and management pose a great challenge for clinicians. It has become widely accepted that inflammation is a driving force behind cancer [3, 4, 27]. However, existing immunotherapies have limited utility in clinical practice [28]. Therefore, there is a need for the development of novel agents with different inflammatory mechanisms to combat diseases.

TMED2 was recently reported to be involved in a variety of tumors, such as multiple myeloma, breast cancer, hepatocellular carcinoma, and choriocarcinoma [9–12]. TMED2 inhibits AGR2 and acts as an inverted systemic alarm signal for pro-inflammatory responses [29]. However, the mechanism underlying the role of TMED2 in inflammation in LUAD is not yet fully understood. In our study, TMED2 expression in LUAD tumor tissues was higher than that in normal tissues, and a higher expression of TMED2 isoforms was associated with a worse prognostic outcome in LUAD patients. These results show that TMED2 may be an oncogene in LUAD and may be related to its prognosis. The GSEA showed that TMED2 expression in LUAD was positively correlated with poor survival and was negatively correlated with apoptosis. These outcomes indirectly proved the above results. As TMED2 was predicted to be an oncogene gene in LUAD via bioinformatics analyses, we detected the difference in protein levels of TMED2 in different tissues and cell lines. The results showed that TMED2 expression was increased in rectal tumor tissue and in HCC827 cells, which were consistent with the results of the bioinformatics analyses.

To further explore the role of TMED2 in LUAD, we performed a lentivirus-mediated knockdown to block the expression of TMED2. In our study, TMED2 knockdown inhibited LUAD development in vitro and in vivo by inhibiting tumor cell proliferation, reducing tumor volume and biomarker levels, and increasing apoptotic levels. These results suggest that TMED2 may regulate tumor development in LUAD and were consistent with the results of the bioinformatics analyses.

Inflammatory molecules may be responsible for increased macrophage recruitment, delayed neutrophil clearance, and increase in reactive oxygen species levels in cancerous tissues [30]. Cytokines and growth factors unusually produced in chronic pulmonary disorders have been found to possess harmful properties that pave the way for epithelial-to-mesenchymal transition and the tumor microenvironment [31]. Inflammation is required for the activation of the TLR4 and NF-κB pathways, which can lead to the expression of pro-inflammatory chemokines [32, 33]. Nevertheless, in LUAD, the mechanism of TMED2 in inflammation via the TLR4/NF-κB signaling pathway is not yet fully understood. In our study, GSEA showed that TMED2 levels were negatively correlated with inflammation, TLR4, and NF-κB. To further investigate the relationship between TMED2 and the TLR4/NF-κB signaling pathway, we used a TLR4 activator, sparstolonin B, to activate the expression of TLR4. The results showed that sparstolonin B increased the levels of TLR4, NF-κB-p-p65, IL-1β, and IL-18 after sh-TMED2 administration in vitro and in vivo. Furthermore, sparstolonin B did not significantly alter the level of TMED2, suggesting that TLR4 may be a downstream factor of TMED2. These results demonstrate that TMED2 may be an upstream target of TLR4 and may possibly be able to regulate inflammation in LUAD through the TLR4/NF-κB signaling pathway.

In further bioinformatics analyses of TMED2 via the GO, KEGG, and the target gene system, we observed that TMED2 was positively or negatively correlated with TME, immune score, TME-associated immune cells, their target markers, and some mechanisms and pathways. Unfortunately, these results which were only based on bioinformatics predictions and analyses could not be explained and verified in this article; it would need to be further investigated thoroughly in the future. We just further propose a possible deeper function of TMED2. Besides, TMED2 may also be related to mutations in TGM4, SPON1, and PLVAP, among other genes, and is also associated with LUAD prognosis. These outcomes require further verification in subsequent studies.

## Conclusions

Our findings indicated that TMED2 knockdown significantly inhibited the development of LUAD by increasing apoptosis, inhibiting tumor cell proliferation, reducing tumor volume, and decreasing the levels of tumor biomarkers and inflammatory factors by affecting the TLR4/NF-κB signaling pathway. Taken together, these findings may provide a new strategy for the treatment of lung carcinoma by regulating inflammation.

## Data Availability

The datasets used and/or analyzed during the current study are available from the corresponding author on reasonable request.

## References

[1] Denisenko TV, Budkevich IN, Zhivotovsky B (2018). Cell death-based treatment of lung adenocarcinoma. Cell Death Dis..

[2] Isla D, de Castro J, Garcia-Campelo R (2020). Treatment options beyond immunotherapy in patients with wild-type lung adenocarcinoma: a Delphi consensus. Clin Transl Oncol..

[3] Wang Q, Li M, Yang M (2020). Analysis of immune-related signatures of lung adenocarcinoma identified two distinct subtypes: implications for immune checkpoint blockade therapy. Aging (Albany NY)..

[4] Jones NL, Xiu J, Rocconi RP, Herzog TJ, Winer IS (2020). Immune checkpoint expression, microsatellite instability, and mutational burden: Identifying immune biomarker phenotypes in uterine cancer. Gynecol Oncol..

[5] Zhou W, Chen X, Hu Q, Chen X, Chen Y, Huang L (2018). Galectin-3 activates TLR4/NF-kappaB signaling to promote lung adenocarcinoma cell proliferation through activating lncRNA-NEAT1 expression. BMC Cancer..

[6] Gong WJ, Liu JY, Yin JY (2018). Resistin facilitates metastasis of lung adenocarcinoma through the TLR4/Src/EGFR/PI3K/NF-kappaB pathway. Cancer Sci..

[7] Barr FA, Preisinger C, Kopajtich R, Korner R (2001). Golgi matrix proteins interact with p24 cargo receptors and aid their efficient retention in the Golgi apparatus. J Cell Biol..

[8] Luo W, Wang Y, Reiser G (2007). p24A, a type I transmembrane protein, controls ARF1-dependent resensitization of protease-activated receptor-2 by influence on receptor trafficking. J Biol Chem..

[9] Ge X, Jiang W, Jiang Y, Lv X, Liu X, Wang X (2020). Expression and importance of TMED2 in multiple myeloma cells. Cancer Manag Res..

[10] Lin X, Liu J, Hu SF, Hu X (2019). Increased expression of TMED2 is an unfavorable prognostic factor in patients with breast cancer. Cancer Manag Res..

[11] Liu Y, Qin Z, Cai L, Zou L, Zhao J, Zhong F (2017). Selection of internal references for qRT-PCR assays of human hepatocellular carcinoma cell lines. Biosci Rep..

[12] Zakariyah A, Hou W, Slim R, Jerome-Majewska L (2012). TMED2/p24beta1 is expressed in all gestational stages of human placentas and in choriocarcinoma cell lines. Placenta..

[13] Li T, Fu J, Zeng Z, Cohen D, Li J, Chen Q, Li B, Liu XS (2020). TIMER2.0 for analysis of tumor-infiltrating immune cells. Nucleic Acids Res..

[14] Huang Z, Lai H, Liao J, Cai J, Li B, Meng L, Wang W, Mo X, Qin H (2021). Upregulation of ADAM12 is associated with a poor survival and immune cell infiltration in colon adenocarcinoma. Front Oncol..

[15] Stadler ZK, Maio A, Chakravarty D, et al. Therapeutic implications of germline testing in patients with advanced cancers. J Clin Oncol. 2021:JCO2003661. 10.1200/JCO.20.03661. 10.1200/JCO.20.03661 PMC8376329 34133209

[16] Zhang A, Yang J, Ma C, Li F, Luo H (2021). Development and validation of a robust ferroptosis-related prognostic signature in lung adenocarcinoma. Front Cell Dev Biol..

[17] Nagy Á, Győrffy B (2021). muTarget: a platform linking gene expression changes and mutation status in solid tumors. Int J Cancer..

[18] Wang JL, Lan YW, Tsai YT (2021). Additive antiproliferative and antiangiogenic effects of metformin and pemetrexed in a non-small-cell lung cancer xenograft model. Front Cell Dev Biol..

[19] Li GZ, Deng JF, Qi YZ, Liu R, Liu ZX (2020). COLEC12 regulates apoptosis of osteosarcoma through Toll-like receptor 4-activated inflammation. J Clin Lab Anal..

[20] Liu D, Gunther K, Enriquez LA (2020). ROR1 is upregulated in endometrial cancer and represents a novel therapeutic target. Sci Rep..

[21] Ma M, Chai K, Deng R (2021). Study of the correlation between the noncanonical pathway of pyroptosis and idiopathic inflammatory myopathy. Int Immunopharmacol..

[22] Jiang L, Niu W, Zheng Q (2021). Identification of an autoantibody against ErbB-3-binding protein-1 in the sera of patients with chronic hepatitis B virus infection. Front Immunol..

[23] Chu Q, Wang S, Jiang L (2021). Patulin induces pyroptosis through the autophagic-inflammasomal pathway in liver. Food Chem Toxicol..

[24] Liu Q, Chen L, Liang X, et al. Exercise attenuates angiotensin-induced muscle atrophy by targeting PPARgamma/miR-29b. J Sport Health Sci. 2021. 10.1016/j.jshs.2021.06.002. 10.1016/j.jshs.2021.06.002 PMC9729927 34116237

[25] Wang Z, Xu J, Wang Y, Xiang L, He X (2021). Total saponins from Tupistra chinensis baker inhibits growth of human gastric cancer cells in vitro and in vivo. J Ethnopharmacol..

[26] Sun GZ, Zhao TW (2019). Lung adenocarcinoma pathology stages related gene identification. Math Biosci Eng..

[27] Bai B, Chen H (2021). Metformin: a novel weapon against inflammation. Front Pharmacol..

[28] Yang X, Zheng Y, Han Z, Zhang X (2021). Functions and clinical significance of KLRG1 in the development of lung adenocarcinoma and immunotherapy. BMC Cancer..

[29] Maurel M, Obacz J, Avril T (2019). Control of anterior GRadient 2 (AGR2) dimerization links endoplasmic reticulum proteostasis to inflammation. EMBO Mol Med..

[30] Pullamsetti SS, Kojonazarov B, Storn S (2017). Lung cancer-associated pulmonary hypertension: role of microenvironmental inflammation based on tumor cell-immune cell cross-talk. Sci Transl Med..

[31] Cho WC, Kwan CK, Yau S, So PP, Poon PC, Au JS (2011). The role of inflammation in the pathogenesis of lung cancer. Expert Opin Ther Targets..

[32] Wang Y, Zeng Y, Zhu L (2020). Polysaccharides from Lentinus edodes inhibits lymphangiogenesis via the toll-like receptor 4/JNK pathway of cancer-associated fibroblasts. Front Oncol..

[33] Lv YX, Pan HR, Song XY, Chang QQ, Zhang DD (2021). Hedyotis diffusa plus Scutellaria barbata suppress the growth of non-small-cell lung cancer via NLRP3/NF-kappaB/MAPK signaling pathways. Evid Based Complement Alternat Med..

